# An Artificial Intelligence System for Staging the Spheno‐Occipital Synchondrosis

**DOI:** 10.1111/ocr.70018

**Published:** 2025-09-02

**Authors:** Omid Halimi Milani, Lauren Mills, Amanda Nikho, Marouane Tliba, Halil Ayyildiz, Veerasathpurush Allareddy, Rashid Ansari, Ahmet Enis Cetin, Mohammed H. Elnagar

**Affiliations:** ^1^ Department of Electrical and Computer Engineering University of Illinois Chicago Chicago Illinois USA; ^2^ Department of Orthodontics, College of Dentistry University of Illinois Chicago Chicago Illinois USA; ^3^ Department of Periodontics, College of Dentistry University of Illinois Chicago Chicago Illinois USA; ^4^ Department of Oral and Maxillofacial Radiology, Faculty of Dentistry Kutahya Health Sciences University Kutahya Turkey; ^5^ Department of Orthodontics, Faculty of Dentistry Tanta University Tanta Egypt

## Abstract

**Objective:**

The aim of this study was to develop, test and validate automated interpretable deep learning algorithms for the assessment and classification of the spheno‐occipital synchondrosis (SOS) fusion stages from a cone beam computed tomography (CBCT).

**Study Design:**

The sample consisted of 723 CBCT scans of orthodontic patients from private practices in the midwestern United States. The SOS fusion stages were classified by two orthodontists and an oral and maxillofacial radiologist. The advanced deep learning models employed consisted of ResNet, EfficientNet and ConvNeXt. Additionally, a new attention‐based model, ConvNeXt + Conv Attention, was developed to enhance classification accuracy by integrating attention mechanisms for capturing subtle medical imaging features. Laslty, YOLOv11 was integrated for fully‐automated region detection and segmentation.

**Results:**

ConvNeXt + Conv Attention outperformed the other models and achieved a 88.94% accuracy with manual cropping and 82.49% accuracy in a fully automated workflow.

**Conclusion:**

This study introduces a novel artificial intelligence‐based pipeline that reliably automates the classification of the SOS fusion stages using advanced deep learning models, with the highest accuracy achieved by ConvNext + Conv Attention. These models enhance the efficiency, scalability and consistency of SOS staging while minimising manual intervention from the clinician, underscoring the potential for AI‐driven solutions in orthodontics and clinical workflows.

## 1 Introduction

In orthodontics, determining growth potential or lack thereof plays a critical role in treatment planning. Certain advantageous orthopaedic movements are only possible when growth potential exists or significantly declines. Accordingly, it is essential to consistently and accurately assess an individual's growth status, and this cannot rely on chronological age alone. Indeed, chronological age has demonstrated upwards of a 2‐year discrepancy with skeletal age, with confounding factors such as sex hormones, genetics and obesity [1, 2, 3]. Biological indicators instead, such as those identified in the hand‐wrist and cervical vertebrae maturation (CVM) methods, are more reliable means of assessing skeletal maturation and consequently routinely used by orthodontists. A common imaging modality in orthodontics is the cone‐beam computed tomography (CBCT), and with the growing desire to reduce radiation, operators can elect to narrow a CBCT field of view (FOV). However, a limited FOV may exclude peripheral anatomy such as the vertebrae, thus negating the CVM staging method for maturity assessment. Nevertheless, there still exists a structure located above the cervical spine that can be employed.

The spheno‐occipital synchondrosis (SOS) is said structure and found in the clivus within the posterior cranial base, just anterior to the foramen magnum [4]. It is the last of three total synchondroses in the cranial base to ossify, accommodating post‐natal brain growth [5]. As the sphenoid and basilar portion of the occipital bone fuse, the cranial base demonstrates elongation and flection at this site, allowing both an increase in facial height and facial depth [6, 7]. This synchondrosis as a result encourages maxilla movement downward and forward, having an impact on the positions of both the maxilla and mandible [8, 9]. The synchondrosis bridges to the maxilla by means of the pterygoid plates, and they are indeed in such close proximity that the width of the synchondrosis itself and the cranial base angle have shown to increase after rapid expansion of the maxilla [10, 11]. The significance of the SOS in ontogeny of the skull is prominent, such that patients with syndromes, for example, Crouzon, Downs and Apert, demonstrate striking midface hypoplasia coinciding with early fusion of this synchondrosis [12, 13]. Inherently, a site with such governance on jaw relations is extraordinarily valuable to examine in the orthodontic field and, as alluded to earlier, can be recruited to measure skeletal maturity in collaboration with the CVM or in lieu of it.

Orthodontists currently predict puberty and growth spurt using the previously mentioned CVM method. As demonstrated through literature, SOS fusion can serve as an alternative route for skeletal maturity evaluation when the cervical vertebrae have been compromised or excluded from a CBCT. To ensure accuracy, an investigation compared hand‐wrist skeletal maturity indicators (SMI) to SOS fusion degrees and found a significant positive relationship [14]. Additionally, a similar study compared CVM and SOS stages and again discovered high correlation, with puberty onset most likely to occur at CVM Stage 3 and correspondingly SOS Stages 1 and 2 [4]. Hence, both investigations concluded that SOS ossification is a reasonable biological indicator for skeletal maturation. Unlike the CVM method, however, SOS staging has yet to be automated to the knowledge of these authors [15]. For the benefit of operator use, it would be of utmost value to develop a model that enables this process to be more efficient, streamlined and dependable.

Artificial intelligence (AI) was founded in the 1950s and has henceforth become integrated across many platforms along with a recent boom in computing power and big data availability. Machine learning (ML) falls within AI and refers to the computer's ability to learn without being explicitly programmed. Deep learning (DL) is yet another subset of ML and involves networks capable of unsupervised learning from unstructured data. Inspired by human neurons, DL has grown popularity in the medical field and has been implemented for image pattern recognition and classification [16, 17]. The application of DL to study SOS fusion is promising and demands a specialised model design adaption and preprocessing technique catered to medical images, which are often associated with subtlety and noise. With this model, clinicians would find more ease and accuracy in diagnosing skeletal maturity, predicting growth potential and prescribing appropriate treatment.

The objective of this paper is to develop, test and validate automated interpretable deep learning algorithms for the assessment and classification of the spheno‐occipital synchondrosis fusion stages from a CBCT.

## 2 Materials and Methods

### 2.1 Preparing the Data Set

In this research, we conducted a retrospective study utilising archived data. All data used in the study was deidentified to ensure confidentiality. The University of Illinois Chicago's Office for the Protection of Research Subjects (OPRS) Institutional Review Board (IRB) approved and provided IRB‐exempt status to the study with the assigned Study ID: STUDY2022‐1048. All methods were performed according to the University of Illinois Chicago's OPRS and IRP guidelines and regulations. The CBCT scans were collected from several private offices in the midwestern United States. The various office machines were from the 17‐19 i‐CAT System with scan parameters set at 120 kv, 4–8 s of exposure time and 0.25–0.4 mm voxel size. The following inclusion criteria were applied: patients had assumed a natural head position with the Frankfort horizontal plane parallel to the ground, jaws were immobilised using a chin holder, teeth were occluded in intercuspal position with facial muscles relaxed, and operators had used an extended field of view (FOV). The exclusion criteria included syndromes affecting the craniofacial and cervical column structures, history of trauma to the head and neck, and previous report of head and neck surgery. This sample consisted of 1200 scans of individuals (592 females, 447 males, 161 unknown) with ages ranging from 7 to 76 years old. Images with synchondroses found in unclear liminal stages, for example, early fusions with faint endocranial borders or late fusions with indiscriminate scar lines, were removed, narrowing the sample to 723 scans (370 females, 260 males, 93 unknown) with ages ranging from 7 to 68 years old. See Tables 1 and 2 for scan distribution per fusion stage and per age cohort, respectively.

**TABLE 1:** Scans per stage.

Fusion stage	Number of scans
1	159
2	92
3	92
4	125
5	255
Total	723

**TABLE 2:** Scans per age cohort.

Age cohort	Number of scans
Child (5–12 years)	281
Adolescent (13–19)	168
Adult (20–39)	116
Middle age (40–59)	55
Senior adult (60+)	9
Unknown	94

Classification was performed by trained evaluators, including two orthodontists and an oral and maxillofacial radiologist. The five‐stage classification system was employed from Bassed et al. (Stages 1–5), with fusion initiating at the superior border (endocranial) and proceeding downward to the inferior (ectocranial) [17]. In Stage 1, the synchondrosis is completely open and unfused. In Stage 2, the superior (endocranial) border has fused while the remainder of the fusion site remains patent. In Stage 3, half the length of the synchondrosis is closed. In Stage 4, closure is essentially complete through the inferior (ectocranial) border, but the site is still visible by a fusion scar. And in Stage 5, the site has been completely obliterated with the appearance of normal bone throughout. To investigate inter‐rater reliability, a crosstabulation was computed using 97 scans. Cronbach's alpha measures the internal consistency of a scale variable and was computed to assess the test‐retest reliability of the SOS maturation stages between two investigators. The alpha was 0.945. It indicates that the inter reliability (> 0.80) is very good.

**FIGURE 1:**
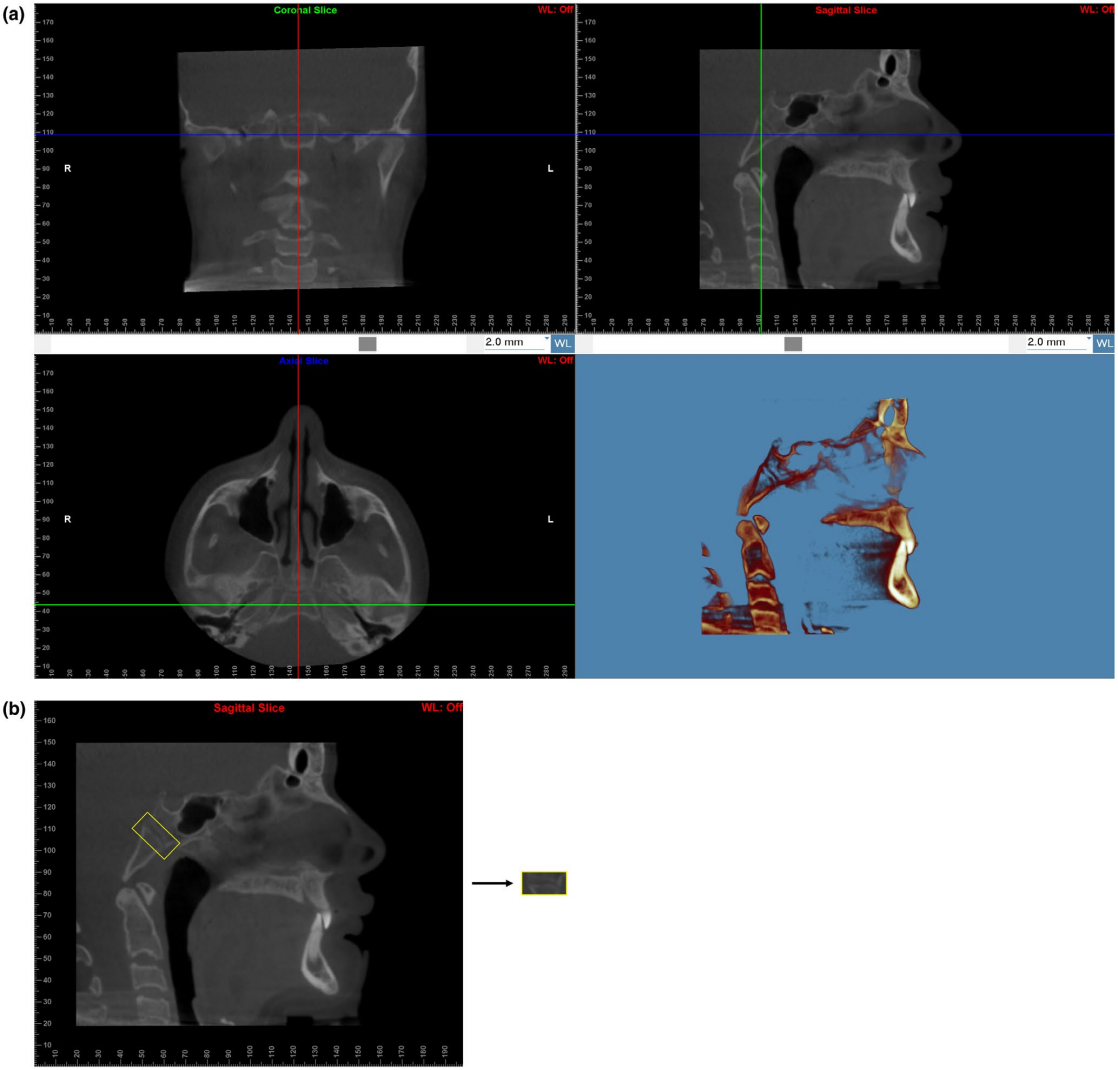
(a) Skull in standard orientation in three planes. (b) Spheno‐occipital synchondrosis rotated and cropped.

The scans were imported in Digital Imaging and Communications in Medicine format into Dolphin Imaging (version 11.95), and their voxel sizes were standardised through resampling to a resolution of 0.5 mm. Orientation of the 3D model was done in the 4‐Equal‐Slices‐Volume‐Layout view and completed in the following manner (Figure 1a). In the coronal view, the axial plane was set parallel to the inferior border of the basilar part of the occipital bone by tilting the head; then, the axial plane was scrolled to its mid‐vertical. In the axial view, the coronal plane was set parallel to the anterior border of the basilar part of the occipital bone by rotating the head; then, the coronal plane was scrolled to its mid‐depth. In both the coronal and axial views, the sagittal plane was scrolled to evenly bisect the basilar part of the occipital bone.

From the sagittal view alone, the Microsoft Snipping Tool (Version 11) was then used to screen capture the entire sagittal slice. The capture was saved as a portable network graphic (PNG) and staged by our observers. Following, the region of interest was extracted from the sagittal image using Microsoft Photos. The synchondrosis was rotated approximately 45° counterclockwise until its ectocranial and endocranial ends lied parallel to the horizontal plane. Then it was cropped at a standardised dimension of 2 × 1 (length by height) (See Figure 1b).

### 2.2 Deep Learning Models for Automated Classification

With the dataset carefully curated and preprocessed to ensure consistency and reliability, the next step involves leveraging advanced deep learning models for automated classification. By utilising pre‐trained architectures known for their robustness in image analysis, we aim to enhance classification accuracy and efficiency while minimising the need for extensive newfound model training. The ResNet family is one of the most widely used architectures in computer vision tasks. It employs residual connections to address the vanishing gradient problem, which enables the training of very deep networks. Among the ResNet models, ResNet18, with 18 layers, is one of the shallowest, yet it still provides solid performance on various classification tasks. As the depth increases, such as in ResNet34, performance improves, with deeper architectures offering greater capacity to learn complex features; though, they require more computational resources [18].

Another model of interest is EfficientNet, a convolutional neural network (CNN) design that merges the strengths of ResNet and MobileNet while addressing their shortcomings [19]. Additionally, ConvNeXt is a modernised version of the traditional CNN that integrates principles from transformer architectures, including attention mechanisms and layer normalisation, while retaining the core advantages of convolutions. The ConvNeXt model, trained on the large ImageNet22k dataset, has demonstrated superior performance in image classification tasks [20]. Furthermore, the ConvNeXt + Conv Attn model utilises convolutional layers with grouped heads to process queries, keys and values, focusing on local patterns rather than global dependencies. By employing element‐wise attention and efficient grouped convolutions, this model is particularly well‐suited for tasks that emphasise local context, while also being computationally efficient for long sequences.

In the convolutional self‐attention mechanism, the queries, keys and values are transformed using one‐dimensional convolutions instead of simple linear transformations, such as those found in traditional multi‐head attention. This convolution operation introduces local receptive fields, enabling the model to capture short‐range dependencies more efficiently. After the convolution, the attention mechanism proceeds by scaling the query, computing attention scores, applying softmax and finally calculating the weighted sum of values, as is done in traditional attention mechanisms.

Furthermore, YOLOv11 represents the latest advancement in the YOLO series, offering improved accuracy and efficiency for real‐time object detection and segmentation. In our work, YOLOv11 is utilised to automate the end‐to‐end pipeline, significantly reducing the operator's workload. By leveraging its precise detection capabilities, YOLOv11 identifies regions of interest (ROIs) within the input data, segments these regions and seamlessly passes them to a classification model for analysis. This level of performance is achieved through several key architectural innovations that refine feature extraction, attention mechanisms and computational efficiency. One of the most significant improvements in YOLOv11 is the Spatial Pyramid Pooling—Fast (SPPF) module, which enhances the model's ability to capture fine‐grained details across different object sizes. Instead of relying solely on fixed receptive fields, SPPF applies multi‐scale pooling, allowing the model to recognise objects at various scales more effectively while keeping computational costs low. This ensures that the network remains lightweight and efficient while still maintaining high detection accuracy.

Another crucial enhancement in YOLOv11 is the Cross Stage Partial with Spatial Attention (C2PSA) module, which introduces a refined spatial attention mechanism. Unlike traditional models that distribute focus evenly across the entire image, C2PSA enables the network to prioritise the most relevant regions, making detection more precise, especially for small or occluded objects. By enhancing the model's ability to distinguish key areas, C2PSA improves both detection speed and reliability in complex visual environments.

Additionally, YOLOv11 incorporates the C3K2 block, an optimised variation of the Cross Stage Partial (CSP) bottleneck. This modification replaces large convolution operations with a more efficient design that utilises smaller, strategically placed kernels. As a result, the model maintains high accuracy while significantly reducing inference time. This improvement not only speeds up processing but also ensures that YOLOv11 remains one of the fastest object detection models available, making it ideal for real‐time applications.

**FIGURE 2:**
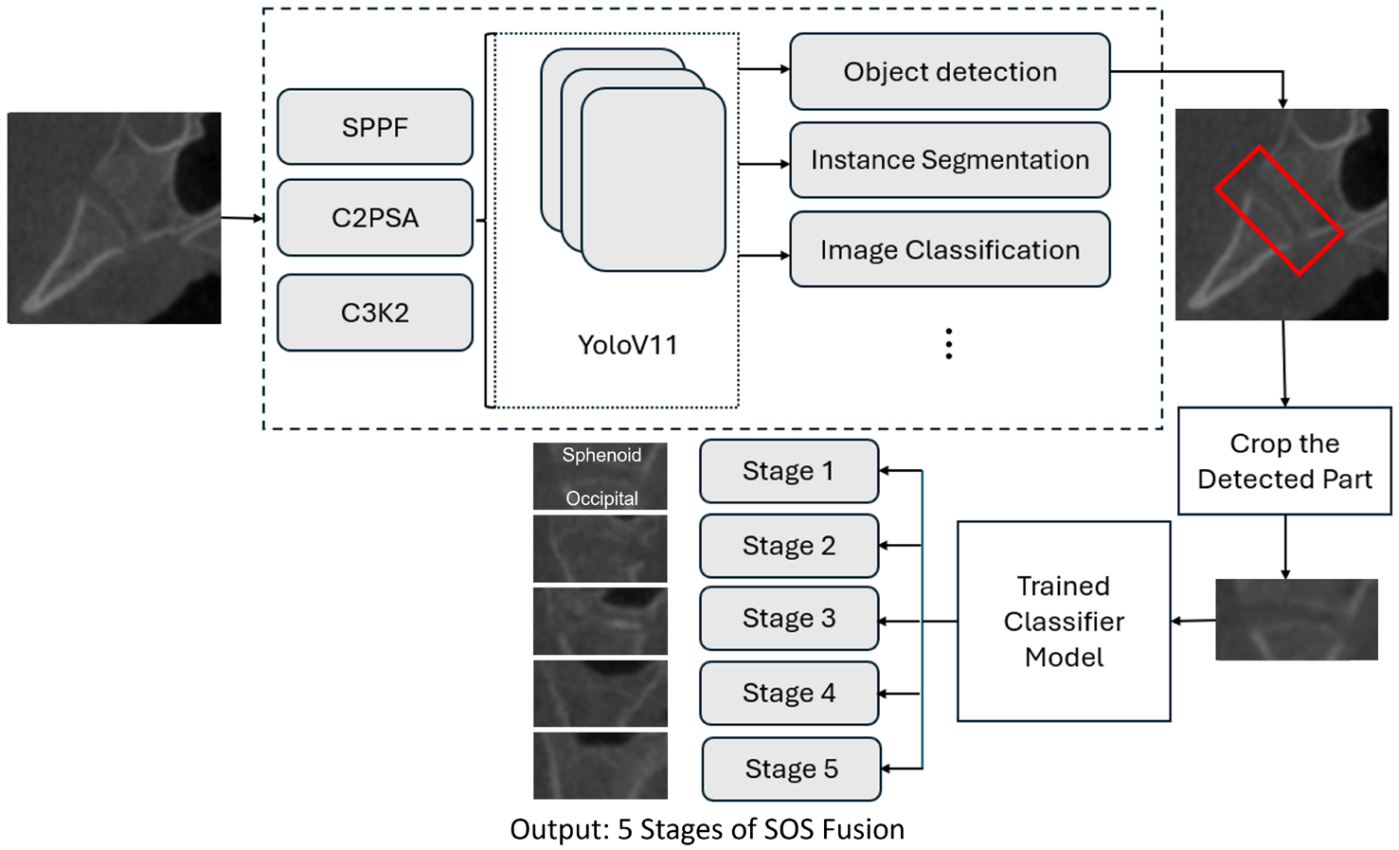
Automated end‐to‐end pipeline: Region detection, cropping and classification.

By integrating these advanced techniques, YOLOv11 optimises computational efficiency while enhancing object detection and segmentation performance. These architectural refinements make YOLOv11 a powerful, scalable and fully automated solution, capable of reducing human effort while maintaining exceptional precision in complex visual tasks [21] (Figure 2).

## 3 Results

The results highlight an important trade‐off between accuracy and automation. To ensure robustness and generalizability, we employed 5‐fold cross‐validation, where the dataset was split into five subsets, using four folds for training (80%) and one fold for testing (20%) in each iteration. When the ROI is manually cropped, the classification models perform slightly better, with the ConvNeXt + Conv Attention achieving the highest accuracy of 88.94% and an F1 score of 88.86%. However, when we introduce automation using YOLO to handle region cropping, there is a small drop in performance, with an accuracy of 82.49% and an F1 score of 82.34%. This drop can be attributed to minor noise or inaccuracies introduced from automated cropping, as it lacks the precision of a human expert carefully selecting the most relevant areas.

Automation, despite causing a slight reduction in accuracy, plays a critical role in enabling scalable and fully autonomous systems. By removing manual intervention, it streamlines workflows, allowing models to handle large‐scale tasks more efficiently. Our YOLOv11 model demonstrates reliable detection performance, achieving a mAP@0.5 of 72.8%. Minor variations in the detected regions had minimal impact on classification results, with the pre‐trained classifier maintaining a solid accuracy of 82.49% ± 3.68% on YOLO‐cropped inputs.

As shown in Table 3, ConvNeXt‐based models consistently delivered the best performance. ConvNeXt + Conv Attn achieved the highest cross‐validation accuracy at 88.94% ± 1.95%, followed by ConvNeXt + Attn (88.24% ± 2.74%) and ConvNeXt (87.39% ± 2.26%). Among the ResNet family, ResNet18 led with 84.87% ± 2.83%, slightly ahead of ResNet34 (84.59% ± 2.60%) and ResNet50 (84.03% ± 1.81%). EfficientNet_b0 showed the lowest overall performance, with an accuracy of 78.57% ± 2.40%.

**TABLE 3:** Performance comparison of models based on Accuracy, Precision, Recall, F1 Score and AUC.

Model	Accuracy (%)	Precision (%)	Recall (%)	F1 (%)	AUC (%)
EfficientNet_b0	78.57	78.48	78.57	77.73	94.34
ResNet18	84.87	84.51	84.87	84.34	96.29
ResNet34	84.59	84.40	84.59	84.14	95.99
ResNet50	84.03	83.95	84.03	83.33	96.06
ConvNeXt	87.39	87.48	87.39	87.24	96.94
ConvNeXt + Attn	88.24	88.93	88.24	88.09	95.18
ConvNeXt + Conv Attn	88.94	89.20	88.94	88.86	96.68
YOLO + ConvNeXt + Conv Attn (Auto Crop)	_82.49_	82.63	82.50	82.34	94.11

**FIGURE 3:**
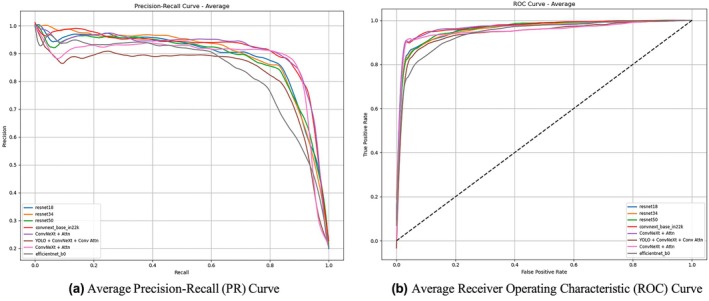
Comparison of (a) PR and (b) ROC curves averaged across all models.

These results highlight the strength and stability of ConvNeXt‐based models across folds, particularly when enhanced with convolutional attention. While automation introduces a small trade‐off in accuracy, its advantages in speed, consistency and scalability—especially for high‐throughput environments like clinical workflows—make it a worthwhile and practical choice for real‐world deployment. Figure 3 illustrates the average Precision‐Recall (PR) and Receiver Operating Characteristic (ROC) curves across all evaluated models, highlighting their classification performance under different decision thresholds.

**FIGURE 4:**
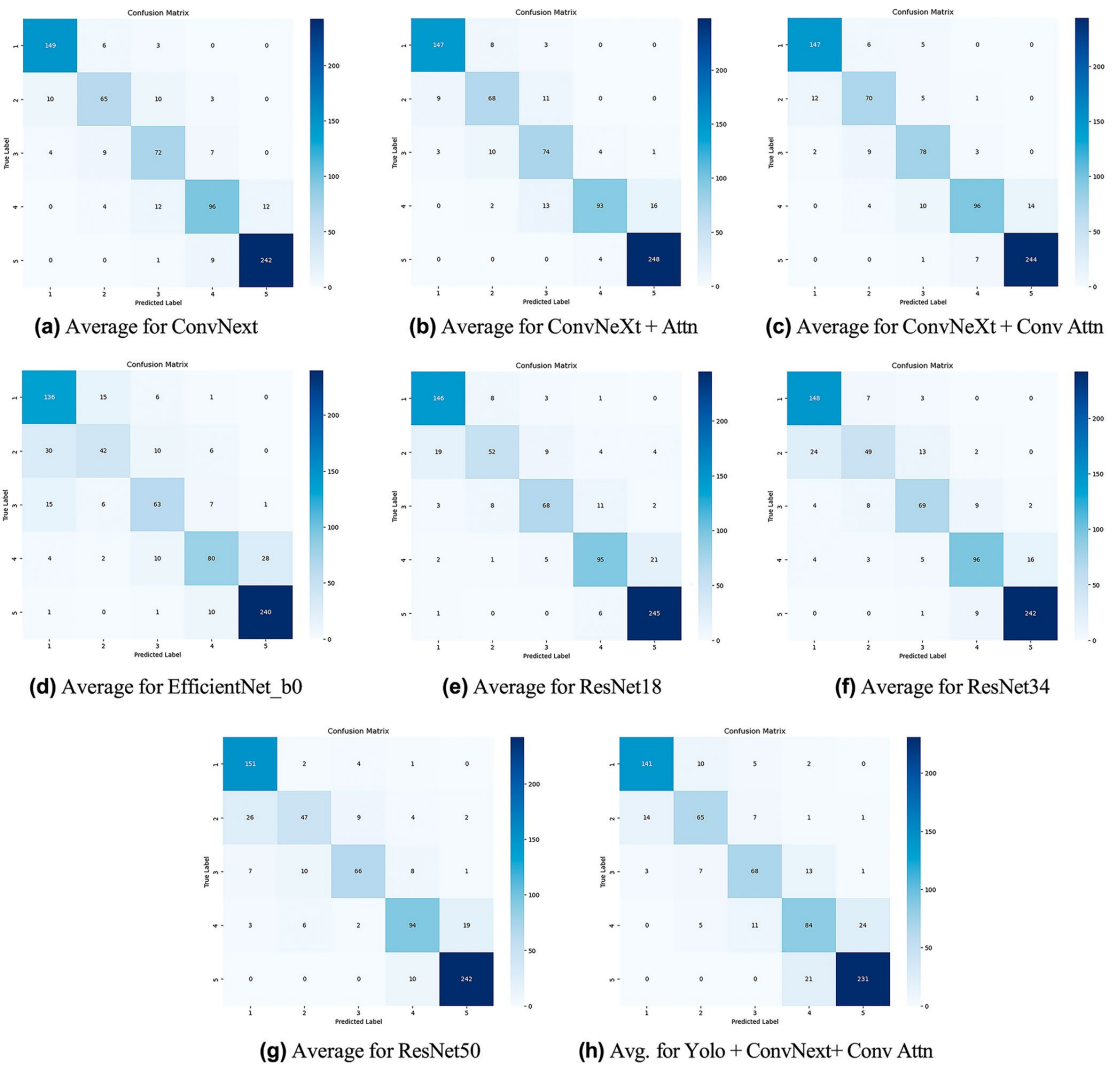
Illustration of the average confusion matrix for various models.

**FIGURE 5:**
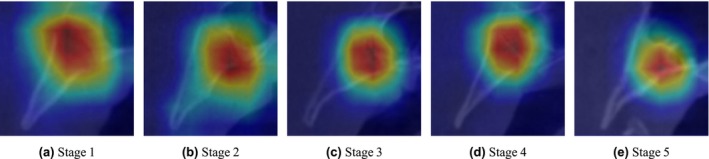
Gradient attention maps generated using Grad‐CAM for a representative sample across all five skeletal maturity stages.

To further investigate the model's behaviour across different skeletal maturity stages, we report detailed stage‐specific metrics in Table 4. This comparison includes precision, recall and F1‐score for each model across all five stages. The results reveal that performance varies by stage and architecture. For instance, while ConvNeXt‐based models consistently perform well, Stage 2 poses the greatest challenge across all models, likely due to subtle anatomical differences at the endocranial border (Figure 4). Notably, the YOLO‐assisted ConvNeXt+ConvAttn model maintains competitive performance, particularly in Stage 3 and Stage 5, suggesting that automated cropping improves the model's focus. Furthermore, to visually interpret model decision‐making, Figure 5 shows Grad‐CAM attention heatmaps for representative samples from each stage. These heatmaps highlight the discriminative regions utilised by the model, offering insights into the anatomical cues leveraged during classification and demonstrating consistent attention to clinically relevant structures.

**TABLE 4:** Stage‐specific performance (Precision, Recall, F1‐score) across different models and stages.

Stage	Metric	EffNet_b0	ResNet18	ResNet34	ResNet50	ConvNeXt	+Attn	+ConvAttn	YOLO + ConvNeXt + ConvAttn
1	Precision	73.89	85.58	82.42	80.93	91.44	92.66	91.38	89.77
	Recall	86.09	92.46	93.69	95.56	94.31	93.08	93.10	89.27
	F1‐Score	79.19	88.86	87.66	87.57	92.84	92.81	92.16	89.37
2	Precision	64.91	75.20	73.50	73.47	76.95	79.11	78.91	74.35
	Recall	47.71	59.02	55.62	53.53	73.66	77.06	79.41	73.46
	F1‐Score	53.94	65.69	63.22	60.80	75.08	77.28	79.05	73.17
3	Precision	70.16	80.16	75.73	81.53	74.16	73.61	79.37	75.16
	Recall	68.60	73.98	75.03	71.64	78.13	80.58	84.91	74.09
	F1‐Score	69.15	76.87	75.30	76.08	75.73	76.55	81.82	74.57
4	Precision	77.86	81.17	83.15	80.72	83.69	92.49	89.88	69.96
	Recall	64.40	76.63	77.33	75.70	77.40	74.97	77.40	67.73
	F1‐Score	69.71	78.66	79.82	77.78	80.11	82.19	83.01	68.47
5	Precision	89.40	90.24	93.17	91.83	95.39	93.71	94.68	90.05
	Recall	95.25	97.24	96.04	96.05	96.05	98.43	96.85	91.67
	F1‐Score	92.18	93.53	94.56	93.83	95.65	95.97	95.70	90.77

## 4 Discussion

Automated staging of the SOS ossification can play a critical role in many fields of study, a novel feat to the knowledge of these authors. Forensic anthropologists, for instance, employ this synchondrosis with its relatively late fusion to perform age estimation in young adults [7, 22]. Meanwhile, orthodontists, as alluded to earlier, can exploit its ossification for skeletal maturity evaluation during orthopaedic treatment planning. Investigations have even identified and linked particular synchondrosis stages to peak mandibular and maxillary growth, providing an avenue for intermaxillary relationship prediction [23, 24]. Nevertheless, its practicality extends beyond pubertal growth assessment.

The SOS fusion state has demonstrated significant positive correlation with the ossification of the zygomaxillary suture, a known obstacle against palatal expansion and can thereby be used to estimate success of rapid maxillary expansion (RME) [8, 25]. RME has also been affiliated with SOS opening and subsequent cranial base angle widening, possibly contributing to midface translation downward and forward [11]. Hence, its fusion may impact the treatment of class III, i.e., midface‐deficient, individuals. This leaves practitioners with the challenge of staging uniformity, which has proven in the literature to be inconsistent in descriptions and methodologies [7].

One of the earliest works defining stages was completed with cephalometric laminography by Powell and Brodie in 1962 [5]. They noted six distinct phases of ossification. Henceforth, multiple different philosophies have been proposed with varying number of stages, definitions and means of assessment. Total number of stages has spanned from three [14, 22], four [9, 23, 24], five [4, 8, 17], and six [5, 6, 26]. Definitions have been as simplistic as unfused, fusing or fused through direct visualisation and as intricate as nodule density evaluation through CT inspection of both axial and sagittal slices [6, 22]. The techniques themselves have also ranged from dry bone, wet bone, histological sections, conventional radiography, CT scans and CBCT scans [22]. This level of incongruity between investigations has consequently led to discourse regarding its validity as an age indicator.

Previous research has proposed that SOS ossification occurs approximately at 18 years old, thereby signifying the age of adulthood for anthropologists [27, 28]. However, that has come to question along with the development of enhanced acquisition means. For instance, a synchondrosis may appear closed when visualised directly but may appear open when viewed with a high‐resolution CT scan. After conducting a literature review, Krishan and Kanchan concluded that its complete ossification is not a reliable indicator for adulthood considering the wide range of reported ages, i.e., from 11 to 25 years [7, 17]. Conversely, there does exist points of mutual agreement among studies. Many authors, for example, link the onset of SOS ossification to the onset of puberty and have correlated its fusion progression with markers of adolescence, such as menarche [4, 9, 22].

Overall, a consensus has been made that findings originating from different methodologies will differ themselves; therefore, the implementation of such findings should be limited to investigations with parallel methods [7]. Hence, the authors of this study employ the staging technique as described by Bassed et al. through use of 3D scans [17]. It should be noted that the majority of radiographic studies diagnose the SOS fusion from two‐dimensional analysis of the midsagittal plane, as done in our study. This plane best visualises the SOS ossification because the sphenoid and occipital bones fuse in the vertical direction from superior to inferior. Though, it is noteworthy that Lottering et al. uniquely referenced the axial plan to supplement their diagnosis, an approach our study did not incorporate, bearing one of the limitations of our work [6]. To enhance accuracy in future applications, our model has the potential to assess ossification stages using the midsagittal slice in conjunction with slices positioned 0.5 mm laterally on either side of this plane to diagnose the SOS fusion.

Furthermore, an additional limitation of our work is in regards to the documented ages and sexes of the scanned individuals. This information was not evaluated for association with specific stages of fusion, as it was not the focus of this study. Future research, though, can delve into the correlation between chronological age and extent of ossification. Many other studies have found sexual dimorphism in fusion, with females reaching complete closure 2–4 years prior to males [5, 22], yet other studies have found insignificant differences between the sexes after 16 years of age [17]. Our dataset is robust in age variety and holds potential to contribute greatly to this discussion.

Reflecting on the objective of this study, the trained models have successfully met our hypothesis and demonstrate accurate staging of the SOS fusion. Noteworthy, while automation is extraordinarily valuable, this tool is not intended to be used in lieu of human expertise, but rather as an adjunct to assist orthodontists, anthropologists and other relevant professionals in their fields of study. The models developed by these authors augment the professional's armamentarium, complementing their proficiency with additional perspective, and can eventually contribute to an AI support system to assist with diagnosis and treatment planning. Our comparative analysis of various state‐of‐the‐art models underscores the trade‐offs between accuracy, computational efficiency and the need for automation. Each model offers unique advantages, and their selection must align with the specific objectives of the task.

The ResNet family, particularly ResNet18 and ResNet34, demonstrated solid performance due to their residual connections, which address the vanishing gradient problem and enable efficient training of deeper networks. However, as the depth increases, these models require greater computational resources, making them less suitable for real‐time applications or resource‐constrained environments. EfficientNet, known for its compound scaling and balanced optimization of depth, width and resolution, provides a more lightweight alternative [19]. Nonetheless, the accuracy of EfficientNet in our experiments was slightly lower compared to deeper architectures, such as ConvNeXt.

ConvNeXt and its variations with attention mechanisms (ConvNeXt + Conv Attn) achieved the highest accuracy and F1 scores. This can be attributed to their integration of modern transformer‐inspired features, such as layer normalisation and attention mechanisms, which enhance the model's ability to capture complex patterns in medical images. The ConvNeXt + Conv Attn model, with its localised attention mechanism, is particularly well‐suited for the subtle and localised variations present in SOS fusion stages, making it an ideal choice for high‐accuracy applications.

Despite the superior performance of these models, the manual cropping of the ROI during classification remains a significant bottleneck. To address this limitation, we integrated YOLOv11, a transformer‐based real‐time object detection model, into our workflow. YOLOv11 precise ROI detection capabilities streamline the end‐to‐end pipeline by automating the cropping and segmentation processes. This automation significantly reduces the time and effort required for manual intervention and ensures consistency across large datasets.

However, the integration of YOLOv11 comes with a modest trade‐off in accuracy. The automated cropping introduces minor noise or misalignment compared to manual cropping performed by experts, resulting in a slight decrease in classification accuracy (from 88.94% to 82.49%). While this reduction is notable, it is outweighed by the operational benefits of automation, particularly in scenarios requiring high throughput or scalability.

The use of YOLOv11 highlights the growing importance of transformer‐based models in medical imaging tasks. Their ability to process and prioritise critical regions aligns well with the objectives of SOS staging, where precise localization and segmentation are paramount. Although there is room for improvement in automated cropping accuracy, future refinements to the YOLO pipeline, such as fine‐tuning on domain‐specific data or integrating additional pre‐processing steps, can help close this gap while retaining the benefits of automation.

Overall, our findings emphasise the need for a balanced approach when selecting models for SOS fusion stage classification. While high‐performing models like ConvNeXt + Conv Attn are indispensable for achieving peak accuracy, the inclusion of automation tools like YOLOv11 is crucial for practical deployment in clinical workflows. By combining these models, we demonstrate the feasibility of a fully automated system for skeletal maturity assessment, paving the way for broader adoption of AI‐driven solutions in orthodontics.

## 5 Conclusion

This work introduces a fully automated pipeline for classifying the spheno‐occipital synchondrosis (SOS) fusion stages, leveraging state‐of‐the‐art deep learning models. By integrating ConvNeXt + Conv Attention with YOLOv11 for precise region detection and segmentation, we achieved a practical balance between accuracy and scalability, marking an advancement in orthodontic diagnostics. The pipeline demonstrates an 88.94% accuracy with manual cropping and maintains commendable performance at 82.49% accuracy with automation, highlighting its robustness and clinical relevance. These achievements pave the way for efficient, scalable and consistent skeletal maturity assessments. Future efforts will focus on utilising knowledge distillation to develop lightweight models capable of high accuracy without relying on YOLO.

## Author Contributions

O.H.M. and M.T. conducted the experiment and wrote the manuscript. R.A. and A.E.C. contributed to the study design and editing of the manuscript. V.A. edited the manuscript. L.M. and A.N. labelled and prepared data and wrote the manuscript. M.H.E. contributed to conception, funding acquisition, study design, data preparation, project director and manuscript writing.

## Consent

All the authors gave full consent for publication.

## Conflicts of Interest

The authors declare no conflicts of interest.

## Data Availability

The data that support the findings of this study are available on request from the corresponding author. The data are not publicly available due to privacy or ethical restrictions.
